# Visualisation and Quantitative Analysis of the Rodent Malaria Liver Stage by Real Time Imaging

**DOI:** 10.1371/journal.pone.0007881

**Published:** 2009-11-18

**Authors:** Ivo H. J. Ploemen, Miguel Prudêncio, Bruno G. Douradinha, Jai Ramesar, Jannik Fonager, Geert-Jan van Gemert, Adrian J. F. Luty, Cornelus C. Hermsen, Robert W. Sauerwein, Fernanda G. Baptista, Maria M. Mota, Andrew P. Waters, Ivo Que, Clemens W. G. M. Lowik, Shahid M. Khan, Chris J. Janse, Blandine M. D. Franke-Fayard

**Affiliations:** 1 Department of Medical Microbiology, Radboud University Nijmegen Medical Centre (RUNMC), Nijmegen, The Netherlands; 2 Unidade de Malária, Instituto de Medicina Molecular, Universidade de Lisboa, Lisboa, Portugal; 3 Leiden Malaria Research Group, Department of Parasitology, Centre for Infectious Diseases, Leiden University Medical Center, Leiden, The Netherlands; 4 Division of Infection and Immunity, Institute of Biomedical Life Sciences & Wellcome Centre for Molecular Parasitology, Glasgow Biomedical Research Centre, University of Glasgow, Glasgow, Scotland; 5 Department of Endocrinology, Leiden University Medical Center, Leiden, The Netherlands; INSERM U567, Institut Cochin, France

## Abstract

The quantitative analysis of *Plasmodium* development in the liver in laboratory animals in cultured cells is hampered by low parasite infection rates and the complicated methods required to monitor intracellular development. As a consequence, this important phase of the parasite's life cycle has been poorly studied compared to blood stages, for example in screening anti-malarial drugs. Here we report the use of a transgenic *P. berghei* parasite, *Pb*GFP-Luc_con_, expressing the bioluminescent reporter protein luciferase to visualize and quantify parasite development in liver cells both in culture and in live mice using real-time luminescence imaging. The reporter-parasite based quantification in cultured hepatocytes by real-time imaging or using a microplate reader correlates very well with established quantitative RT-PCR methods. For the first time the liver stage of *Plasmodium* is visualized in whole bodies of live mice and we were able to discriminate as few as 1–5 infected hepatocytes per liver in mice using 2D-imaging and to identify individual infected hepatocytes by 3D-imaging. The analysis of liver infections by whole body imaging shows a good correlation with quantitative RT-PCR analysis of extracted livers. The luminescence-based analysis of the effects of various drugs on *in vitro* hepatocyte infection shows that this method can effectively be used for *in vitro* screening of compounds targeting *Plasmodium* liver stages. Furthermore, by analysing the effect of primaquine and tafenoquine *in vivo* we demonstrate the applicability of real time imaging to assess parasite drug sensitivity in the liver. The simplicity and speed of quantitative analysis of liver-stage development by real-time imaging compared to the PCR methodologies, as well as the possibility to analyse liver development in live mice without surgery, opens up new possibilities for research on *Plasmodium* liver infections and for validating the effect of drugs and vaccines on the liver stage of *Plasmodium*.

## Introduction

Malaria remains a major cause of global morbidity and mortality. New anti-malarial drugs are urgently needed, especially with the increase in drug resistant parasites and the lack of effective vaccines and vector control measures [1]–[4]. The main site for intracellular development of human and rodent *Plasmodium* sporozoites after they are injected by an infected mosquito is the liver. This stage of the parasite's development is clinically silent and therefore regarded as an ideal point of intervention for prophylactic or vaccine strategies [5]–[7]. The liver stage of *Plasmodium*'s life cycle has also received particular attention in the context of *P. vivax*, the second most important agent of human malaria, which can generate cryptic forms called hypnozoites that persist in the liver for long periods of time [8]–[10]. These dormant forms of the parasite are responsible for what is termed relapsing malaria, which may occur following latent periods of months or even years without new infection [10], [11]. In comparison with drugs that kill blood stage parasites, only a limited number of drugs exist that act on liver stages; most notable amongst these are primaquine, atovaquone and tafenoquine [12], [13], and only primaquine [14], [15] has been shown to act on the hypnozoite stage of *P. vivax* [14], [15]. Clearly, the development of new inhibitors/drugs against the malaria liver stage would target an important and under-exploited site of intervention [1], [16].

Quantitative analysis of liver stage *Plasmodium* development both *in vivo* in laboratory rodents and *in vitro* in cultured liver cells is hampered by the low levels of parasite infection and by the complicated methods required to monitor parasite development. As a consequence, the development of novel and efficient methods for analysing/screening the effect of drugs and small molecule inhibitors on the parasite's intracellular growth in the liver lags well behind the more rapid developments being made in the automated drug/inhibitor screening assays for blood stage parasites [17]–[20]. Currently, one of the standard ways to assess drug efficacy against liver stages is to monitor *in vitro* liver stage development by quantitative RT-PCR (qRT-PCR) methods [21]–[23] [24], [25] that are time consuming and expensive. Other studies have involved direct quantification and viability of parasite development by microscopy [26], [27], RNA hybridization [28], or infrared fluorescence scanning system [29]. However, these methods are not only prone to large variations between observers but are also time consuming given the very low infection rates (generally less than 2%) observed in cultured hepatocytes [29]. Moreover, simple and efficient methods for analysing *in vivo* liver stage development in small laboratory animals are completely absent. The recent generation of new transgenic rodent malaria parasites expressing fluorescent reporter proteins has enabled an intimate analysis of *Plasmodium* sporozoites interacting with host hepatocytes during invasion and subsequent development inside hepatocytes, both *in vitro* and *in vivo* [30]–[34]. Recently, GFP-expressing parasites have been used in conjunction with flow cytometry to provide quantitative information on the parasites development in hepatic cells [35]. However, the use of fluorescent parasites in *in vivo* analysis of *Plasmodium* liver stage development requires complex surgery and when such parasites are used in conjunction with flow cytometry, their usefulness is presently restricted to *in vitro* and *ex vivo* analyses.

We have previously reported the use of transgenic *P. berghei* parasites expressing the bioluminescent reporter protein, luciferase, to examine the distribution and development of sequestering blood stage parasites in live animals using real time imaging [36], [37]. Recently, we have also shown the effectiveness of such bioluminescent reporter parasites in simple and sensitive microplate reader assays for screening of drugs against blood stage parasites both *in vitro* and *in vivo* in rodents [19]. For these assays we generated a transgenic *P. berghei* parasite line that expresses a luciferase-GFP fusion protein and is free of a drug-selectable-marker [38]. In the study described here, we utilised the luminescent properties of this reporter parasite, *Pb*GFP-Luc_con_, to analyse liver stage development by real time imaging both in cultured hepatocytes and within the liver of living mice. We established that the changes in bioluminescence are directly proportional to the level of hepatocyte infection *in vitro* and *in vivo*, determined by comparison with standard qRT-PCR methodologies. As the liver parasite infection progresses real-time *in vivo* imaging allows the identification of individual infected hepatocytes in living animals. We demonstrated the application of the technique for the *in vitro* screening of compounds targeting the liver stage and the use of real-time imaging to determine *in vivo* drug sensitivity of liver stages through analysis of the effect of primaquine. Importantly, bioluminescence imaging also allows the course of an infection to be monitored, both throughout liver stage parasite development and in the blood stage of infection without sacrificing the animal, and therefore, can greatly reduce the number of experimental animals required to determine drug sensitivity. Since bioluminescence imaging is relatively simple to execute, the use of the methodologies described in this paper will greatly simplify the analysis of drug toxicity and small molecule inhibition on liver stage parasite growth.

## Materials and Methods

### Experimental animals

Female C57BL/6 and Swiss CD1 mice, 6–8 weeks old (Charles River), weighing 20 to 35 g at the time of primary infection and female Wistar rats (Harlan; 175–200 g) were used.

All studies in which animals were involved have been performed according to the regulations of the Dutch “Animal On Experimentation act” and the European guidelines 86/609/EEG.

### Transgenic parasite line

The transgenic *P. berghei* line 676m1cl1 line (*Pb*GFP-Luc_con_) has been used in this study (mutant RMgm-29 in www.pberghei.eu). It expresses a fusion GFP (mutant 3) and firefly luciferase (LucIAV) and has been generated in the reference clone of ANKA strain cl15cy1 [38]. Parasites of line 676m1cl1 contain the *PbGFP-Luc* gene fusion stably integrated as a single copy gene by double cross over recombination into the *230p* locus and the reporter gene is under control of the constitutive *eef1aα* promoter [39]. This line has been selected by FACS-sorting of GFP-expressing parasites and therefore does not contain a drug-selectable marker. This line can be obtained from the Malaria Research and Reference Reagent Resource Center, MR4 (http://www.malaria.mr4.org).

### Collection of sporozoites

*Anopheles stephensi* mosquitoes were infected by feeding on infected mice using standard methods of mosquito infection. On day 21–28 after infection, the salivary glands of the mosquitoes were collected by hand-dissection. Salivary glands were collected in DMEM (Dulbecco's Modified Eagle Medium from GIBCO) and homogenized in a home made glass grinder. The free sporozoites were counted in a Bürker-Türk counting chamber using phase-contrast microscopy.

### Sporozoites traversal and gliding

Traversal assays were performed as described previously [40]. Briefly, Huh7 cells were plated in 24 well plates (10^4^ cells/ml) and an equivalent number of sporozoites was added to the wells with the addition of FITC labeled dextran (Invitrogen, NL). No sporozoites were added to the negative control wells that were used as threshold for the FACS analysis. FACS analysis was performed on 25 000 cells per well (wells were prepared in triplicate) using a FACScalibur flowcytometer (Becton Dickinson, NL).

Gliding assays were performed in precoated (3D11, 10 ug/ml) Labtek slides (Nunc, NL) and 2×10^4^ sporozoites were added. After 30 minutes of incubation at 37°C sporozoites were fixed with 4% PFA and after washing with PBS, the sporozoites and the trails (‘gliding circles’) were stained with 3D11-Alexa 488 conjugated antibody (Dylight 488 antibody labelling kit; Thermo Scientific, NL). Slides were mounted with Fluoromount-G (SouthernBiotech, NL) and ‘gliding circles’ were analyzed using a Leica DMR fluorescence microscope at ×1000 magnification.

### *In vitro* development of liver stages in hepatocyte cultures

To measure the luciferase activity of liver stages in HepG2 cells, a total of 2×10^4^ to 1.5×10^5^ sporozoites were added to monolayers of 2×10^5^ HepG2 cells (1 ml/well in 24 well plates) as described previously [41]. Cells were prepared in quadruplet wells. In several assays, Cytochalasin D (Sigma, NL) was added to the cells at a concentration of 10 µg/ml prior to addition of the sporozoites as previously described [42]. At different time points after invasion, 100 µl of cells were collected, transferred to 96-well plates and processed for imaging with the Lumina system (see below). Four hundred µl of the remaining cells were harvested and lysed with either 200 µl of RLT buffer (RNA easy kit, Quiagen, NL) or 200 µl of cell culture lysis reagent obtained from the Promega Luciferase Assay System Kit® (Promega, NL) and stored at −80°C until further analysis by qRT-PCR or bioluminescence with a microplate reader(see below).

To measure the luciferase activity of liver stages in Huh7 cells a total of 5×10^3^ to 7×10^4^ sporozoites were added to triplicate wells containing monolayers of 7×10^4^ Huh7 cells (400 µl/well in 24 well plates) as previously described [35]. At different time points after sporozoite addition, cells were harvested and lysed with either 150 µl of qRT-PCR buffer (RNA easy kit, Quiagen, NL) or 100 µl of cell culture lysis reagent obtained from the Promega Luciferase Assay System Kit® (Promega, PT). Samples in Promega lysis buffer were either stored at −80°C or processed immediately to measure luminescence intensity with the Lumina system (see below) or bioluminescence analysis by microplate reader (see below) and qRT-PCR samples were stored at −80°C until further analysis by qRT-PCR analysis (see below).

### Real time measurements of bioluminescence of *in vitro* cultured liver stages using the Lumina system

The *in vivo* imaging system Lumina (Caliper Life Sciences, USA) was used to measure luciferase activity of infected HepG2 and Huh7 cells. Imaging data were analysed using the Living Image® 3.0 software (Caliper Life Sciences, USA). For the infected HepG2 cells, 100 µl of Assay Substrate (Promega Luciferase Assay System Kit®) were added to 100 µl of hepatocyte cultures collected in 96-well plates (see above) and bioluminescence images were acquired with a 12,5 cm field of view (FOV), medium binning factor and an exposure time of 1 to 3 minutes. For infected Huh7 cells, 70 µl of Luciferase Assay Substrate (Promega Luciferase Assay System Kit®) were added to 20 µl of lysed hepatocyte cultures in black 96-well plates. Bioluminescence images were acquired with a 12,5 cm FOV, medium binning factor and an exposure time of 5 minutes.

### Bioluminescence measurements of *in vitro* cultured liver stages using a microplate reader (luminometer)

For infected HepG2 cells, 100 µl of Luciferase Assay Substrate (Promega Luciferase Assay System Kit®) were added to 10 µl of lysed parasite samples in 96-well plates. Luminescence spectra of the samples were measured using a microplate reader (Wallac 1420 multilabel counter, PerkinElmer, NL) and the light reaction of each well was measured for 10 s. Measurements of luciferase activity are expressed as relative luminescence units (RLU). For infected Huh7 cells, 75 µl of Luciferase Assay Substrate (Promega Luciferase Assay System Kit®) were added to 15 µl of lysed parasite samples in white 96-well plates. Luminescence intensity of the samples was measured using a microplate reader (Tecan, CH) and the light reaction of each well was measured for 5 seconds. Measurements of luciferase activity are expressed as relative luminescence units (RLU).

### *In vivo* development of liver stages in mice

Mice were inoculated with sporozoites by i.v. injection of 1×10^3^, 1×10^4^, 5×10^4^ or 1×10^5^ purified sporozoites or by mosquito bite (5–10 infected mosquitoes per mouse) at day 20–22 after the infectious blood meal. Blood stage infections were monitored by analysis of Giemsa-stained blood smears of tail blood collected on day 4–10 after inoculation of sporozoites or infection by mosquito bite.

### Real time *in vivo* imaging of liver stage development in whole bodies of live mice or in dissected livers

Luciferase activity in animals was visualized through imaging of whole bodies or of dissected livers using the *in vivo* Imaging System (IVIS 100 and Spectrum; Caliper Life Sciences, USA) as described in Franke-Fayard *et al.* [37]. Animals were anesthetized using the isofluorane-anesthesia system (XGI-8, Caliper Life Sciences, USA), their belly was shaved and D-luciferin dissolved in PBS (100 mg/kg; Synchem Laborgemeinschaft OHG, Germany) was injected subcutaneously (in the neck). Animals were kept anesthetized during the measurements, which were performed within 3 to 5 minutes after the injection of D-luciferin. Bioluminescence imaging was acquired with a 10 cm FOV, medium binning factor and an exposure time of 10 to 180 seconds.

Luciferase activity in individual livers was visualized in whole organs dissected 44 h after sporozoite injection or mosquito bite. Livers were obtained by dissection of animals 2 to 3 min after a second intravenous injection of D-luciferin (in the tail vein; 100 mg/kg). Livers were placed in Petri-dishes or on black tape to minimize light interference from plastic Petri-dishes. Dissected livers were imaged with a 10 cm FOV, medium binning factor and an exposure time of 60 to 180 seconds. Imaging data were analysed using the Living Image® 3.0 (Caliper Life Sciences, USA) software.

Quantitative analysis of bioluminescence of whole bodies or dissected livers was performed by measuring the luminescence signal intensity using the ROI settings of the Living Image® 3.0 software. The ROI was set to measure either the abdominal area at the location of the liver for whole body imaging or the complete livers in the case of dissected livers. ROI measurements are expressed in total flux of photons.

For the 3D imaging of luciferase activity in live mice, the *in vivo* imaging system IVIS® 3D (Caliper Life Sciences, USA) was used as described [43]–[45]. The IVIS® 3D performs rotational axis imaging of the bioluminescent light sources within a living animal. The IVIS 3D acquires eight imaging views about the longitudinal axis of the animal at 3 different wavelengths: 580, 600 and 620 nm. At each angle view, the animal height or surface topography is determined and stitched together to generate the whole 3D map of the animal. The 3D diffuse tomography software (Living Image ^TM^) is used to reconstruct the eight bioluminescent images resulting in data on *in vivo* source brightness, location, and size of the infection. Exposure time was of 60 seconds for each angle of measurements. A digital female mouse atlas was overlaid onto the 3D diffuse tomography reconstruction to obtain anatomical reference points. This feature is included in the Living Image Software 3D Analysis Package. The liver was removed from the 3D reconstruction of the mouse organs to better visualize the bioluminescence signals.

### Analysis of *in vitro* development of liver stages in hepatocyte cultures and in extracted livers by qRT-PCR

RNA was extracted from hepatocyte culture samples collected in 200 µl (HepG2) or 150 µl (Huh7) of qRT-PCR buffer (see above) with Quiagen's MicroRNeasy kit following the manufacturer's instructions. The transcriptor first-strand cDNA synthesis kit (Roche) was used according to the manufacturer's recommendations to make single-stranded cDNA. RNA was extracted from livers collected at 44 h after infection and homogenized in RLT buffer (DNA/RNA Quiagen extraction kit) supplemented with 0,07% β-mercaptoethanol and stored at −80°C till qRT-PCR analysis. The RNA samples were further processed as described above for the samples of the hepatocyte cultures.

Real time PCR analysis of specific *P. berghei* parasite 18S rRNA and β actin mouse (HepG2 invasion) or Hypoxanthine Guanine Phosphoribosyl Transferase (HPRT; Huh7 invasion and whole infected livers) housekeeping genes was done according to [21], [35]. Standardization was done by multiplying the value of each sample with a correction factor. This correction factor is the maximum value for the housekeeping genes found for all samples divided by the value of this gene obtained for the sample).

### Analysis of drug-inhibition of *in vitro* liver stage development

For the analysis of inhibition of *in vitro* liver stage development by drugs, 3×10^4^ sporozoites were added to monolayers of 7×10^4^ Huh7 cells (400 µl/well) in 24 well plates as described above. Five drugs that are known to inhibit liver stage development were used to test the drug susceptibility: primaquine (primaquine diphosphate 98%, Aldrich, NL); tafenoquine (GlaxoSmithKline, UK); genistein [25]; lopinavir [24] and saquionovir [24]). Primaquine was dissolved in water to a final stock solution of 100 µM and serial dilutions with complete culture medium were prepared ranging from 1 µM to 100 µM. Tafenoquine was dissolved in ethanol to a final stock concentration of 100 µM and serial dilutions were prepared ranging from 0,3 to 30 µM. Genistein, lopinavir and saquinavir were dissolved in water to a final stock concentration of 100 µM, 100 µM and 25 µM, respectively. Serial dilutions with complete culture medium were prepared, ranging from 10 to 100 µM for genistein and 2,5 to 40 µM for lopinavir and saquinovir. Huh7 cells were incubated with different concentrations of the drugs in triplicate wells by replacing the culture medium with drug-containing medium prior to sporozoite addition. Forty-six hours after adding the sporozoites, the infected Huh7 cells were harvested and lysed with 100 µl of cell culture lysis reagent obtained from the Promega Luciferase Assay System Kit®. Seventy-five µl of Luciferase Assay Substrate (Promega Luciferase Assay System Kit®) were added to 15 µl of lysed parasite samples in white 96-well plates. Luminescence spectra of the samples were measured using a microplate reader (Tecan, CH) and the light reaction of a sample of each well is measured for 5 seconds. Measurements of luciferase activity are expressed as relative luminescence units (RLU).

### Analysis of the inhibition of *in vivo* liver stage development by primaquine and tafenoquine

Mice were treated with primaquine (primaquine diphosphate 98%, Aldrich, NL) and tafenoquine (GlaxoSmithKline, UK) once at day −1, twice on the day of infection (day 0; 5 hours before and after infection) and once the following day (day +1; 19 h and 29 h after infection). Both primaquine and tafenoquine were dissolved in distilled water and administered subcutaneously with concentrations ranging from 1–40 mg/kg body weight and 10 and 20 mg/kg body weight respectively. Mice were infected at day 0 by the bite of 5–10 mosquitoes, as described above. *In vivo* imaging was performed at 44 hours after infection as described above. At day 6 – 9 after infection, the same mice were analysed for blood stage infections by determination of the course of parasitemia in Giemsa stained thin blood films of tail blood.

### Growth inhibitory curves and statistical analysis

The two tailed analysis using the Spearman's rho test of the SPSS 16 software (SPSS Inc., USA) was used for statistical analysis. Correlation coefficients were determined using the two-tailed Spearman's rho test for non-parametric analysis of small data set. qRT-PCR curves were drawn using the GraphPad Prism software (GraphPad Prism, Inc., US). p values were calculated using the same GraphPad Prism software. The non-linear regression function for sigmoidal dose-response (variable slope) of the GraphPad Prism software was used to calculate the (best-fit) effective concentration (EC_50_) values.

## Results

### Analysis of *Pb*GFP-Luc_con_ liver-stage development *in vitro*

For the analysis of liver stage development we used a transgenic *P. berghei* parasite, *Pb*GFP-Luc_con_ (line 676m1cl1), which expresses a reporter fusion gene of *gfp* and *luciferase*, stably integrated in the *230p* locus (PB000423.03.0) of the *P. berghei* genome. *Pb*GFP-Luc_con_ parasites do not contain a drug-resistance marker as they were selected by FACS sorting of transfected GFP-positive blood stages immediately after the transfection procedure [38]. The *gfp-luciferase* transgene in *Pb*GFP-Luc_con_ is under the control of the *P. berghei eef1a* promoter. Through the analysis of GFP expression we have previously demonstrated that the *eef1a* promoter drives constitutive and strong gene expression in all life cycle stages, including liver stage parasites [39]. The blood and mosquito stages of *Pb*GFP-Luc_con_ show similar growth characteristics as those of the parent reference line, cl15cy1 of *P. berghei* ANKA (data not shown). Analysis of sporozoite motility, cell traversal and *in vitro* and *in vivo* infectivity demonstrated that all features of *Pb*GFP-Luc_con_ sporozoites were comparable to those of wild type sporozoites (Figure S1).

**Figure 1:**
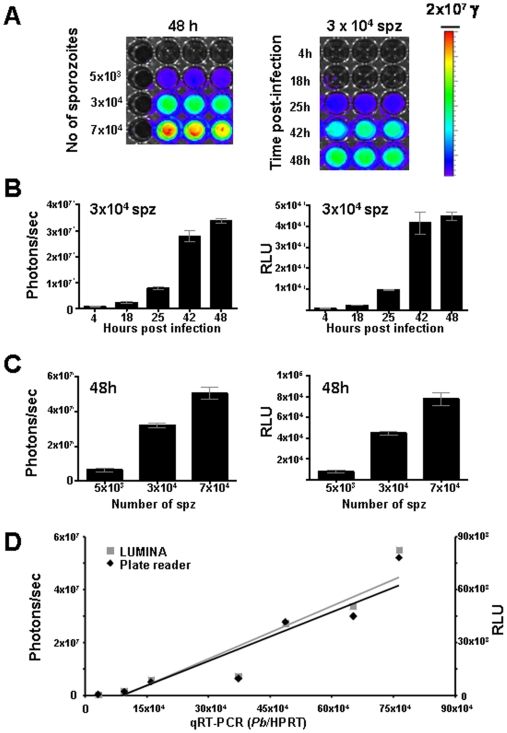
Analysis of *in vitro* liver stage development by determination of luciferase expression (luminescence). A. Luminescence levels (photons/sec) during liver stage development of *Pb*GFP-Luc_con_ after infection of Huh7 cells with different numbers of sporozoites at 48 h (left panel) and at different time points after infection with 3×10^4^ sporozoites (right panel) determined by direct imaging of samples using the Lumina system. Rainbow images show the relative levels of luminescence ranging from low (blue), to medium (green), to high (yellow/red). B. Luminescence levels during development of liver stages at different time points after invasion of Huh7 cells as measured by the Lumina system (Photons/sec) and a Tecan microplate reader (Relative light unit, RLU). C. Relationship between the numbers of sporozoites used to infect Huh7 hepatocyte cultures and the luminescence produced by the liver stages at 48 h after infection. Luminescence levels were determined by direct imaging of samples using the Lumina system (Photons/sec) and a Tecan microplate reader (RLU). D. Correlation between luminescence values as measured by the Lumina system and the Tecan microplate reader and of *P. berghei* 18S rRNA levels as determined by qRT-PCR of Huh7 cultures that are infected with different numbers of sporozoites. See Table S1 for the correlation coefficient data of the two-tailed Spearman's rho test.

To determine the timing and level of luciferase expression of *Pb*GFP-Luc_con_ throughout development of liver stages *in vitro*, two hepatoma cell lines, HepG2 and Huh7, were infected with different numbers of sporozoites, ranging from 5×10^3^ to 1.5×10^5^, in 24-well plates. The time course of luciferase expression during the first 48 hours of development is shown in Figures 1A&B and S2A&B. The luminescence intensity (luciferase activity) was measured by (a) direct imaging of the culture plates of live or lysed cells using the Lumina system (the luminescence intensity expressed as photons per second) or by (b) analysis of lysed cell samples in a microplate reader (luminescence intensity expressed as Relative Light Units, RLU). Both methods show a strong increase in luciferase activity throughout the 48 h period during which the invaded sporozoites develop into liver schizonts. The increase in reporter protein expression during trophozoite and schizont development is expected as a similar increase in *eef1a* based expression of luciferase or GFP is observed in blood stage trophozoites and schizonts [19], [36]. Uninfected control cells showed low photon counts and luminescence values are significantly lower than those of infected cells at any of the time points assessed The mean photon counts were 3×10^6^ p/s (sd 2×10^6^) and 5×10^4^ p/s (sd 1×10^3^) and the mean RLU values were 56 (sd 17) and 30 (sd 15) for HepG2 and Huh7 cells respectively. Sporozoites contain low levels of the GFP-Luciferase protein as shown by analysis of GFP expression by fluorescence-microscopy (data not shown) and therefore low bioluminescence levels at 4–5 h might be derived from invaded sporozoites. A strong increase in luminescence values is observed after 24–30 h which correlates with the development of the liver trophozoite into the schizont stage. For further quantitative analyses of liver stage development we compared luminescence levels of samples taken at time points between 30 and 48 h after sporozoite incubation.

Luminescence intensities at 30 and 48 h correlate well with the number of sporozoites added to the hepatocytes in the range of 5×10^3^ to 1×10^5^, using both the Lumina and the microplate reader (Figures 1C, S2A–C). When using as few as 5×10^3^ sporozoites a clear luminescent signal is obtained that is significantly higher than the background signal detected in uninfected wells (p = 00,1). We then compared the relative luminescence intensities of cells infected with different sporozoite numbers with the relative amounts of parasite 18S ribosomal RNA using standard qRT-PCR methodologies (Figures 1D, S2D). A good correlation was observed between the relative luminescence intensities and the relative amounts of parasite 18S rRNA in the same cultures (Spearman correlation coefficient ranging from 0.61–0.94; Table S1).

### Analysis of *Pb*GFP-Luc_con_ liver-stage development *in vivo*

**Figure 2:**
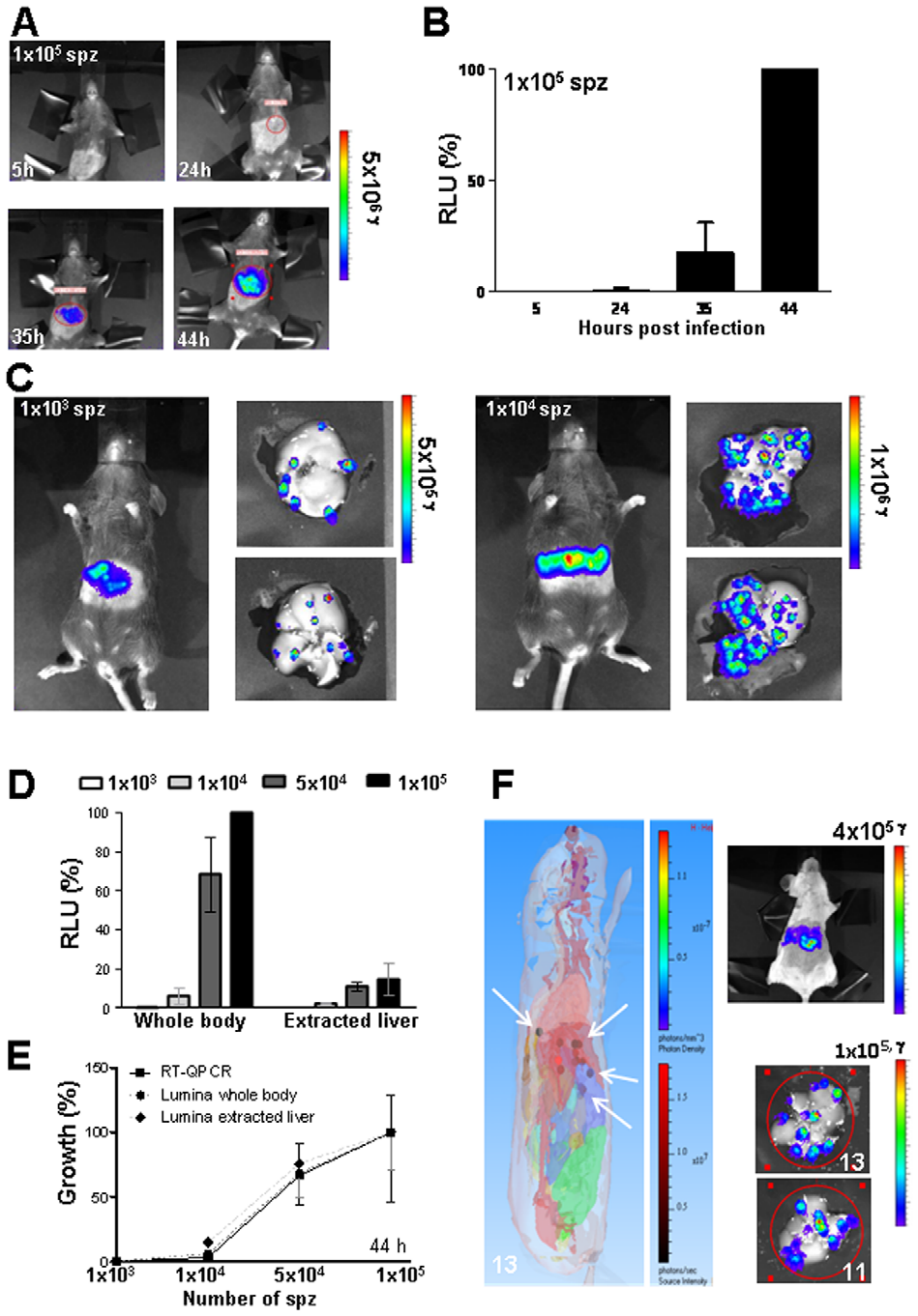
Analysis of *in vivo* liver stage development by determination of luciferase expression (luminescence). A. Representative rainbow images of luminescence in livers of live mice at different time points after injection of 1×10^5^ sporozoites. Rainbow images show the relative levels of luminescence ranging from low (blue), to medium (green), to high (yellow/red). B. Luminescence levels (photons/sec) of livers in whole mice at different time points after infection with 1×10^5^ sporozoites (n = 4). Photon counts from whole body imaging are expressed as the percentage of the photon counts of mice at 44 h after infection ( = RLU %). C. Distribution of luminescence signals in the livers of live mice and in extracted livers of the same mice at 44 h after infection with 1×10^3^ (left) or 1×10^4^ (right) of sporozoites. D. Luminescence levels (photons/sec) of whole bodies and extracted livers of mice 44 h after inoculation of different numbers of sporozoites. Photon counts are expressed as the percentage of the photon counts of whole body of mice at 44 h infected with 10^5^ sporozoites ( = RLU %). E. Correlation between luminescence values as measured by the Lumina system of whole body and dissected livers and of *P. berghei* 18S rRNA levels as determined by qRT-PCR of dissected livers that are infected with different numbers of sporozoites. The percentage of growth is normalized to the highest reading within each experiment. See Table S2 for the correlation coefficient data of the two-tailed Spearman's rho test. F. The left panel shows the 3D-imaging of luminescence signals (3D tomography and source reconstruction) in a mouse at 44 h after infection with 5 to 10 mosquito bites as measured with the IVIS 3D Series system. The brown/red spots (white arrows) indicate the origin of highest luminescence intensity in the body. These spots are located in the liver as shown by overlaying with a digital mouse atlas to obtain anatomical reference points (see also Supplementary Movie S1 of mouse 1). The right panel shows the same mouse and its extracted liver (imaged at both sides) imaged with the 2D-IVIS100 imaging system. Numbers in the images represent the number of luminescent spots identified. The number of spots (13) in the whole body is determined by the 3D analysis as can be seen in Supplementary Movie S1.

To determine the timing and level of luminescence during *Pb*GFP-Luc_con_ development in the liver, groups of mice (n = 4) were infected intravenously with different numbers of sporozoites ranging from 1×10^3^ to 1×10^5^. Luciferase activity in the animals was visualized through the imaging of whole bodies using the IVIS100 imaging system at 5, 24, 35 and 44 hours after infection. In control, uninfected mice, luminescence values ranged between 1×10^7^ and 4×10^7^ p/s (sd 1×10^7^). In mice infected with the highest dose of sporozoites (i.e. 1×10^5^), 3 mice showed luminescence levels above background at 24 h (i.e. 1×10^8^ p/s (sd 3×10^7^); see Figure 2A&B). Mice infected with 5×10^4^ sporozoites showed a signal above background at 35 h. In all infected mice there was a strong increase in bioluminescence signal between 35 and 44 h (Figures 2C, S3) whereas between 44 h and 52 h no further increase was observed and, indeed, in several mice the luminescence signal decreased between these time-points (Figure S4A). After 60 h, luminescence signals could be detected in the whole body, resulting from parasites that had invaded erythrocytes after the rupture of the liver schizonts (Figure S4A). The decrease in luminescence in the liver between 44 and 52 h may either be due to liver schizont rupture and the consequent reduction in the number of infected liver cells or is the results of decrease in luciferase expression in the final stages of schizont maturation. Such a decrease has been previously observed in erythrocytic schizonts where protein expression peaks in mature trophozoites/young schizonts and decreases in maturing schizont when the *eef1a* promoter is used to drive protein expression [36], [39] and correlates with destruction of endogenous eef1a mRNA in schizonts [46]. Based on these observations, we decided to determine luminescence intensities at 44 h in subsequent experiments. When luminescence intensities were measured at 44 h, a good correlation was observed between the luminescence intensity and the number of sporozoites initially injected (Figures 2D&E). Specifically, the mean luminescence intensity of mice infected with 1×10^3^ sporozoites was 1×10^9^ p/s (sd 4×10^8^) and increased to 1×10^10^ p/s (sd 7×10^9^) in mice infected with 1×10^4^ sporozoites.

After the whole body measurements, the livers of several of the mice from each group were dissected and imaged with the IVIS100 system. The luminescence intensity of the extracted livers was significantly lower than that of whole bodies (Figures 2C&D, S3). For example, livers from mice infected with 1×10^5^ sporozoites had, on average, a ten-fold lower luminescence signal compared to whole body imaging (8×10^8^ p/s, sd 4×10^8^; Figure 2D). The presence of clearly separated luminescent spots in dissected livers of mice infected with low numbers of sporozoites (1×10^3^; Figures 2C, S3) indicates that these spots represent individual liver schizonts. Therefore, imaging of dissected livers may provide information on both the number and dissemination of parasites in the liver. When livers containing 3 to 13 individual spots were imaged, both sides often showed a comparable numbers of spots in a similar location (Figures S3B, S4B). However in each liver imaged, one or a few luminescent spots were only visible on one side of the liver, indicating that the imaging of these spots can be influenced by their localization, possibly due to a quenching effect of the liver. To better localize the origin of individual luminescent spots, we used the IVIS 3D Series system (Caliper Life Sciences, USA) to image luminescent signals in live mice in three dimensions. This instrument, in combination with the 3.1 Living Image® software, allows the precise localization of the origin of the luminescent signals in whole bodies in contrast to the more diffuse luminescence signals obtained with the IVIS100 2D-system. 3D-imaging of 4 infected mice in an anatomical context show the presence of clearly separated spots in the liver (Figures 2F and S5). The individual infected hepatocytes can be best visualized in the context of the whole liver when the mice are rotated as visualized in the Supplementary movies S1-S3. When the number of luminescent spots was determined by 2D-imaging in livers dissected after the 3D-imaging of the whole mice, a good correlation between the numbers of spots obtained with both methods was found. These observations indicate that 3D-imaging of whole bodies allows the detection of individual liver schizonts in live mice. However, like in 2D-imaging of isolated livers, some luminescent spots may be missed in the 3D-imaging, as shown in mouse 4 Figure S5.

As described for the *in vitro* analysis of liver stage development, we compared the relative luminescence intensities of whole bodies and isolated livers measured at 44 h pi with 18S ribosomal RNA qRT-PCR data derived from RNA extracted from the same livers. The relative luminescence intensities of whole bodies and dissected livers are in good agreement with the 18S rRNA qRT-PCR values (i.e. Spearman correlation coefficient ranging from 0.65 to 0.95; Figure 2E, Table S2). The best correlation is found between qRT-PCR and whole body imaging, possibly because of the decrease of luminescence during extraction of the livers as discussed above.

Rats (e.g. Sprague-Dawley, Wistar etc) as well as mice are frequently used to analyse liver stage development in the *P. berghei* model of malaria. We have performed a limited number of experiments to investigate whether *in vivo* imaging of liver stage development in Wistar rats generates similar results to the *in vivo* imaging in mice (Figure S4B). In rats luminescence signals were detected at 24 h after infections had been initiated by mosquito bite with rapidly increasing luminescence intensities during the period of 24–30 h. Imaging of dissected livers from these rats also showed the same pattern of clearly separated luminescent spots (on both sides of the liver) as we had observed in extracted mouse livers (Figures 2C, S3).

### Analyses of drug-inhibition of *Pb*GFP-Luc_con_ liver stage development by luminescence measurements

**Figure 3:**
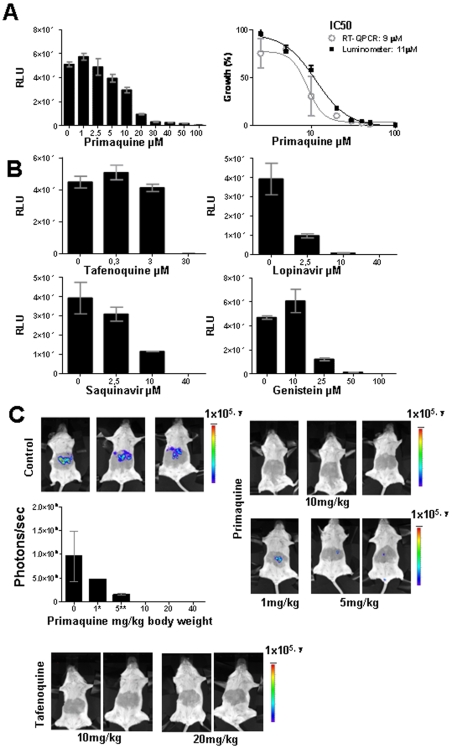
Drug-inhibition of liver stage development determined by measurement of luciferase expression (luminescence). A. Inhibition of *in vitro* liver stage development by primaquine (left panel) by measuring luminescence levels (RLU) in samples of Huh7 cells 44 h after infection of the cells with 3×10^4^ *Pb*GFP-Luc_con_ sporozoites. The right panel shows the inhibition of liver stage development by primaquine as determined by both luminescence measurements and qRT-qPCR analysis. The percentage of growth is defined by the RLU values and by the amounts of *P. berghei* 18S rRNA levels, respectively. Luminescence levels were measured using a Tecan microplate reader. B. Inhibition of *in vitro* liver stage development by tafenoquine, lopinavir, sanquinavir and genistein, as determined by measuring luciferase luminescence levels (RLU) in samples of Huh7-infected cells 44 h after infection of the cells with 3×10^4^ *Pb*GFP-Luc_con_ sporozoites. Luminescence levels were measured using a microplate reader. C. Inhibition of *in vivo* liver stage development by primaquine and tafenoquine as determined by measuring luminescence levels (photons/sec) in live mice at 44 h after infection of the mice by the bite of 5 infected mosquitoes. Luminescence levels were determined by direct imaging of whole bodies using the IVIS100 system.

Having established that liver stage infection can be accurately and conveniently measured *in vitro* and *in vivo* by assessing the luminescence of *Pb*GFP-Luc_con_-infected cells or mice livers, we decided to investigate the suitability of this method for the evaluation of anti-plasmodial drugs. The inhibition of *in vitro* development by drugs was determined by measurement of luminescence of *Pb*GFP-Luc_con_-infected hepatoma cells maintained in 24-well plates and incubated with serial dilutions of five different drugs known to inhibit liver stage development (Figures 3A&B). Primaquine [47] tafenoquine [48] genistein [25], lopinavir [24] and saquinavir [24] were added to Huh7 cells 1 h before addition of Pb-GFP-Luc_con_ sporozoites and luminescence was measured 44 h later with a microplate reader. In samples treated with the highest drug concentrations, known to completely block liver stage development, the luminescence values are low and almost identical to background ranging consistently from 20 to 350 RLU (mean of 104; sd 119). In contrast, in drug-free control samples luminescence values ranged between 4×10^4^ to 7×10^4^ RLU (mean 5×10^4^; sd 9×10^3^) in the different experiments (Figure 3A&B). Primaquine's IC50 value as determined by luminescence intensity correlated well with the value obtained by standard qRT-PCR methods (Figure 3A). Complete inhibition with primaquine and tafenoquine was observed at concentrations of 100 µM and 30 µM, respectively, which correspond to inhibitory concentrations reported in the literature for primaquine (2×10^−7^ to 5×10^−5^ M) and tafenoquine 3×10^−7^ M [23], [47], [49]. Genistein, lopinavir and saquinavir concentrations that inhibited liver stage development, quantified by the decrease in luminescence intensities are also in good agreement with previously reported inhibitory concentrations for these compounds that were determined by direct counting of liver stages or by qRT-PCR (Figure 3B)[24], [25].

Analysis of *in vivo* inhibition of liver stage development by luminescence measurements was performed using primaquine and tafenoquine. Mice were treated 5 times with different doses of these drugs starting one day before infection with *Pb*GFP-Luc_con_ and the last dose at 29 h after infection. Mice were infected by the bites of 5 infected mosquitoes and luminescence levels were determined 44 h later. Luminescence values of untreated, control mice, ranged between 2×10^8^ and 2×10^9^ p/s (mean 1×10^9^; sd 5×10^8^). No detectable luminescence signal was observed in mice treated with 10–40 mg/kg body weight of primaquine, indicating complete inhibition of parasite growth (Figure 3C). Indeed, analysis of these mice 5–9 days after infection showed no detectable parasites in peripheral blood, whereas control mice developed normal blood infections with parasitemias ranging between 0.1 and 3% at day 4 post-infection. In mice treated with 1 and 5 mg/kg body weight of primaquine, 3 out of 6 mice showed a low level of luminescence ranging between 1×10^8^ and 5×10^8^ p/s (mean 3×10^8^; sd 2×10^8^) at 44 h while the remaining 3 mice were negative. Five of these mice developed a blood stage parasitemia that was delayed by two days compared to the control mice (parasitemia of 0,5 to 3% at day 6 after infection), indicating a 100-fold inhibition of liver stage development. All mice treated with 10 or 20 mg/kg of tafenoquine were luminescence negative at 44 h and did not develop blood stage infection (Figure 3C). The complete inhibition of liver stage development by primaquine and tafenoquine at doses of 10 mg/kg body weight and higher is in agreement with the inhibitory doses reported in the literature [25], [50], [51].

## Discussion

Rodent malaria parasites are frequently used for the identification and characterization of new anti-malarial drugs [17]–[20], [25], [50], [52], [53]. These parasites are used in initial drug and small molecule inhibitor (SMI) screens in order to determine their *in vivo* anti-malarial activity in cultured cells and in mice. In comparison to the blood-stage parasite SMI screening assays [19] the screening and identification of agents that inhibit *Plasmodium* development in the liver is considerably more complex. Quantitative analysis of liver stage development both in cultured liver cells, *in vitro*, and in small laboratory animals, *in vivo*, is hampered by the low levels of parasite infection as well as the complicated, time consuming and expensive methods required to monitor parasite development, such as qRT-PCR or direct counting of liver stages [21]–[23], [26], [27] and RNA hybridization [28], [29]. We have recently shown that transgenic rodent parasites expressing luciferase are useful reagents to determine parasite load and bio-distribution of blood stages in live mice using *in vivo* imaging [36], [37]. We have also used these parasites to assess the sensitivity of blood stages to drugs by measuring luminescence using a microplate reader based assay [19]. We now show that luminescence assays can also be used for the quantitative analysis of liver infection and that the results of these assays closely correlate to standard analysis methods (i.e. qRT-PCR). The transgenic parasite used in these assays, *Pb*GFP-Luc_con_, expresses luciferase under the control of the strong and constitutive *eef1a* promoter. This promoter has previously been shown to drive expression of reporter proteins in growing and dividing stages throughout the parasite's life-cycle [39]. The strong increase in reporter gene expression using this promoter from sporozoite-hepatocyte invasion to mature liver schizont is matched with reporter gene expression from merozoite-erythrocyte invasion to schizogony. The significant increase in luminescence 5–10 hours after sporozoite infection of hepatocytes, as compared to cultures incubated with sporozoites whose ability to invade liver cells is impaired (i.e. treated with cytochalasin-D), shows that luciferase production starts rapidly after invasion of the hepatocyte. We reproducibly observed a clear increase in luminescence 48 hours post infection in hepatocyte cultures infected with as few as 5×10^3^ sporozoites, compared to uninfected control wells. This sensitivity of the luminescence assays with low sporozoite numbers in combination with the early detection of luciferase expression offers unique possibilities for large scale screenings of inhibitors of parasite liver stage development, with the potential for automation, using microplate assays. The use of such assays would confer the same advantage currently only available to drug screening against blood stage parasites [19].

Despite the expression of luciferase during the early stages of parasite development within hepatocytes, we were not able to detect luminescence signals in live mice during the first 20 hours of infection, even at the highest infection dose of 1×10^5^ sporozoites. To investigate whether the sensitivity of detection of the young liver stages could be increased, we analysed a transgenic parasite line (mutant RMgm-152 in www.pberghei.eu) that expresses *Pb*GFP-Luciferase under the control of the promoter of the circumsporozoite protein (CS; PB001026.00.0). The sporozoite stage of these reporter parasites strongly expresses the reporter fusion protein as visualised by GFP-fluorescence intensity; but we were still unable to detect sporozoites in the liver by *in vivo* imaging, although we were able to detect sporozoites in the skin at the site of biting when we measured mice directly after mosquito feeding (data not shown). Although we were not able to detect the young liver stages, the more mature liver stages were readily detected 30 h post infection of the mice, even after infection with a sporozoite dose as low as 1×10^3^ sporozoites. The 30–48 h period corresponds to the phase of schizogony during which a single parasite can produce more than 1×10^4^ daughter merozoites [54]. It is known that laboratory mice are relatively insensitive to infection with *P. berghei* sporozoites and therefore the sensitivity of *in vivo* imaging might even be higher if the reporter line were made in another rodent malaria parasite, *P. yoelii*, to which mice are more sensitive. When blood stage infections were analysed in mice that resulted from infections initiated with 1×10^3^ sporozoites we calculated that the luminescence signal measured at 48 h was the result of only 1–5 schizonts. This is based on the assumption that the parasite multiplication rate in erythrocytes is 10-fold every 24 hours [55] and that each liver schizont contains between 2×10^3^ and 1×10^4^ merozoites. The detection of localised spots of luminescence in dissected livers indicates that the *in vivo* imaging enables detection to the level of a single infected hepatocyte containing a mature liver schizont. However, the total luminescent intensity of extracted livers was lower than the luminescence intensity of livers determined by imaging of live mice. This was initially surprising because the expected quenching of luminescence by tissues in live mice would be absent when the isolated organ was examined. However, the lower values obtained from dissected livers are most likely the result of the rapid uptake and possibly metabolism of luciferin [56] during the time required to collect the liver.

It has recently been shown that mature liver schizonts produce so called merosomes, packets of 100–200 merozoites surrounded by the host cell membrane [57], [58]. The possibility to detect bioluminescence signals of individual liver schizonts might also offer opportunities to analyse the process of merosome formation as well as merosome migration after their release from the infected hepatocyte. Merosomes are released in the blood circulating and appear to specifically accumulate in the lungs whereupon they burst open and merozoites are released and invade red blood cells [58]. It would therefore be interesting to see if the methodologies in this study can be adapted to also image the merosomes in the liver and then in isolated lungs or in lungs of whole bodies of animals to add to our understanding of merosome biology.

The similar numbers of luminescence spots detected in dissected livers and in living mice (analysed by 3D imaging) also supports the notion that *in vivo* imaging can detect an individual mature liver schizont. However, in dissected livers there were several luminescence spots that were detected at only on one side of the liver and by combining whole body imaging and imaging of dissected livers we found that a minor fraction of the schizonts was undetectable by either of the two methods. In addition, a few mice treated with non-curative doses of primaquine showed no luminescence signals but developed a (delayed) blood stage infection. These observations indicate that small numbers of liver schizonts can be missed with whole body imaging, although in the case of primaquine treatment the absence of a luminescence signal might also be due to delayed development of the liver schizonts. To investigate whether we could increase the sensitivity of detection of mature liver schizonts we have separately analysed a different transgenic line which expresses luciferase under the control of the *ama1* promoter (PB000821.01.0) [36]; mutant RMgm-30 in www.pberghei.eu). The *ama1* gene encodes the micronemal protein, AMA1, in merozoites and it was our contention that since very large numbers of merozoites are produced in each liver schizont we could expect a high luciferase signal. Surprisingly, using similar sporozoite numbers as used with our *ef1aa* promoter line, we measured a significantly lower luminescence signal, even in measurements that were taken at later time points (48–60 h) after infection (data not shown).

The analysis of drug-inhibition of parasite liver stage development by *in vivo* imaging offers clear advantages over standard qRT-PCR analysis of dissected livers or analysing the dynamics of the blood stage infection subsequent to liver infection. qRT-PCR analysis is both time consuming and expensive whereas the analysis of subsequent blood stage infections cannot easily discriminate the effect of the drugs on liver stage and/or resulting blood stage infections. In contrast, *in vivo* imaging is rapid and simple and allows, within the same animal, to measure both the specific inhibition of liver stage development by an inhibitor or drug and its subsequent effects on the blood stages. The analysis by *in vivo* imaging has the advantage in that analysis does not require sacrificing the experimental animal and thereby reduces the number of animals required for experimentation since multiple measurements can be made in the same animal over time. Moreover, it also has the advantage that it minimizes the biological variation within the study [59], [60,60]. The *in vivo* analysis of drug sensitivity of liver stages to primaquine and tafenoquine was performed with mice that were infected by the bite of only five infected mosquitoes. All the control mice in these experiments (i.e. infections in the absence of drug) show a strong luminescence signal at 48 h after infection. These experiments demonstrate that *in vivo* drug-sensitivity assays are not dependent on the injection of mice with high numbers of sporozoites, which requires time-consuming manual dissection from mosquito salivary glands. The sensitivity of *in vivo* imaging therefore greatly simplifies the procedure of *in vivo* drug-sensitivity testing. An additional feature of the reporter protein luciferase that may be of great benefit is that it has a relatively short half-life and therefore only allows the detection of live parasites, thereby avoiding errors potentially associated with the counting of dead liver parasites (as may occur with qRT-PCR experiments). The imaging assays described in this paper can also be used for the screening and analysis of parasite mutants for aberrant liver stage development. Moreover, these can be used to analyse liver stage development in challenge studies of mice that are immunized with either subunit vaccines against sporozoites/liver stage molecules or with genetically attenuated sporozoites. In conclusion, quantitative analysis of liver stage development by real-time imaging should greatly aid the validation of drugs and vaccines that act against the liver stages of the *Plasmodium*.

## Supporting Information

Supplementary Table S1 (0.10 MB DOC)

Supplementary Table S2 (0.06 MB DOC)

Supplementary Figure S1 Analyis of sporozoite motility, cell traversal and infectivity of PbGFP-Luccon A. Representative immunofluorescence staining with anti-PbCSP ([61]) of the trails produced by PbGFP-Luccon (left) and wild type sporozoites (right). Characteristic circles of gliding motility are observed in PbGFP-Luccon sporozoites. B. Cell traversal ability of wild type and PbGFP-Luccon sporozoites as determined by FACS counting of Dextran positive Huh7 cells. FACS counting was performed 3 h after infection of Huh7 cells with 6×104 sporozoites. Uninfected: hepatocytes cultured in the presence of Dextran but without the addition of sporozoites. C. Infection of Huh7 cells on coverslips using 3×104 PbGFP-Luccon (left) and PbGFPcon [39] (right) sporozoites. After fixing and staining, similar numbers of exoerythrocytic forms are observed at 48 h post infection for both parasites. D. qRT-PCR quantification of in vitro invasion of HepG2 cells by wild type and PbGFP-Luccon at 24 h (black bars) and at 45 h post invasion (white bars). Cyto D: cultures with cytochalasin-D. E. qRT-PCR quantification of liver invasion in mice of wild type and PbGFP-Luccon sporozoites. qRT-PCR was performed on material from livers collected at 43 h after infection of the mice with 3×104 sporozoites. The pre-patent period, defined as the days between injection of sporozoites and a blood infection with a parasitemia of 0.5–2%, was 4.2 days (range 4–5 days) for PbGFP-Luccon compared to 4.4 days (range 4–5) for wild type parasites after injection of 1×104 sporozoites. After injection of 1×104 sporozoites the pre-patent periods were 5.3 days (range 5–6) for PbGFP-Luccon and 5.5 days (range 5–6) for wild type parasites.
(0.28 MB TIF)

Supplementary Figure S2 Analysis of in vitro liver stage development in HepG2 cells by determination of luciferase expression (luminescence). A. Relationship between the numbers of sporozoites used to infect hepatocyte cultures and the luminescence produced by the liver stages at 24, 30 and 48 h after infection. Luminescence levels were measured using the Lumina system (Photons/sec). B. Relationship between the numbers of sporozoites used to infect hepatocyte cultures and the luminescence produced by the liver stages at 24, 30 h, 48 h after infection. Luminescence levels were measured using the Lumina system (Photons/sec) and a Wallac microplate reader (Relative light units, RLU), respectively. C. Relationship between the numbers of sporozoites used to infect hepatocyte cultures and the luminescence produced by the liver stages at 30 after infection. Luminescence levels were measured using the Lumina system (Photons/sec) and a Wallac microplate reader (Relative light unit, RLU), respectively. D. Correlation between luminescence values and 18S rRNA levels. Luminescence values were determined using the Lumina system and the Wallac microplate reader (see C). P. berghei 18S rRNA levels were determined by qRT-PCR of hepatocyte cultures infected with different numbers of sporozoites. The percentage of growth is normalized to the highest reading within each experiment. See Table S1 for the correlation coefficient data of the two-tailed Spearman's rho test.
(0.15 MB TIF)

Supplementary Figure S3 Imaging of whole bodies and dissected livers (IVIS100) of mice at 44 h after infection by 1×103 (A) or 1×104 sporozoites (B). Dissected livers were imaged at both sides. Numbers in the pictures of Panel A show the number of luminescent spots identified.
(0.68 MB TIF)

Supplementary Figure S4 A. Whole body imaging (IVIS100) of two representative mice during the period of 24–68 h after infection by bites of 20 infected mosquitoes, showing a strong increase of luminescence intensity of the liver during the period of 30–44 h after infection and a subsequent decrease after 52 h in the liver. The strong increase in luminescence of the whole body at 68 h is the result of the dissemination of the liver merozoites released into the bloodstream and subsequent invasion of erythrocytes. Rainbow images show the relative level of luminescence ranging from low (blue), to medium (green), to high (yellow/red). B. Imaging of whole bodies and extracted livers (IVIS100) of Wistar rats at 44 h after infection by bites of 1 or 5 infected mosquitoes. Extracted livers were measured at both sides (a, b) and lobes (c) and small sliced liver pieces (d) were analysed for additional luminescence spots. Numbers in the images represent the number of luminescent spots identified.
(0.46 MB TIF)

Supplementary Figure S5 A. Source reconstruction of 3D whole body imaging of three mice at 44 h after infection by bites of 5–10 infected mosquitoes. Eleven luminescent sources are detected in mouse 3 (M3), one in mouse 5 (M5) and none in mouse 4 (M4). See also Supplementary Movies 2 and 3 corresponding to mouse 3 and 5 respectively. B. 2D-imaging of the extracted livers of the mice shown in panel A. Livers were imaged at both sides using the IVIS Spectrum system. Numbers in the images represent the number of luminescent spots identified. Rainbow images show the relative level of luminescence ranging from low (blue), to medium (green), to high (yellow/red).
(0.74 MB TIF)

Movie S1 (2.29 MB WMV)

Movie S2 (0.49 MB WMV)

Movie S3 (0.46 MB WMV)
