# Supplementary material for: Visualisation and Quantitative Analysis of the Rodent Malaria Liver Stage by Real Time Imaging
Source: PLoS One. 2009 Nov 18;4(11):e7881. doi: 10.1371/journal.pone.0007881 (PMC2775639; doi:10.1371/journal.pone.0007881)
Supplement: Supplementary Table S1 — (0.10 MB DOC) [file pone.0007881.s001.doc]

**Supplementary Table S1**

Correlation coefficient data (ρ, two-tailed Spearman’s rho test) of the luminescence data (Lumina and Microplate reader) and the RT-qPCR data presented in Figure 1 and S2.

|  |  | RTqPCR | Lumina | Luminometer |
| --- | --- | --- | --- | --- |
|  |  | **HepG2, Ei: 25K, 50K, 75K spz at 24h** | | |
| RTqPCR | ρ | 1,00 | 0,80** | 0,52 |
|  | N | 12 | 12 | 12 |
| Lumina | ρ | 0,80** | 1,00 | 0,61* |
|  | N | 12 | 12 | 12 |
| Microplate reader | ρ | 0,52 | 0,61* | 1,00 |
|  | N | 12 | 12 | 12 |
|  |  | **HepG2, Eii: 25K, 50K, 75K spz at 30h** | | |
| RTqPCR | ρ | 1,00 | 0,88** | 0,92** |
|  | N | 10 | 10 | 10 |
| Lumina | ρ | 0,88** | 1,00 | 0,85** |
|  | N | 10 | 12 | 12 |
| Microplate reader | ρ | 0,92** | 0,85** | 1,00 |
|  | N | 10 | 12 | 12 |
|  |  | **HepG2, Eiii: 25K, 50K, 75K spz at 48h** | | |
| RTqPCR | ρ | 1,00 | 0,83* | 0,78 |
|  | N | 6 | 6 | 6 |
| Lumina | ρ | 0,83* | 1,00 | 0,93* |
|  | N | 6 | 6 | 6 |
| Microplate reader | ρ | 0,78 | 0,93* | 1,00 |
|  | N | 6 | 6 | 6 |
|  |  | **Huh7: 5K, 30K, 75K spz at 48h** | | |
| RTqPCR | ρ | 1,00 | 0,83* | 0,87** |
|  | N | 11 | 8 | 11 |
| Lumina | ρ | 0,83* | 1,00 | 0,95** |
|  | N | 8 | 9 | 9 |
| Microplate reader | ρ | 0,87** | 0,95** | 1,00 |
|  | N | 11 | 9 | 12 |
|  |  | **Huh7: 4, 18, 25, 42 hpi; 30K spz** | | |
| RTqPCR | ρ | 1,00 | 0,94* | 0,98* |
|  | N | 11 | 11 | 11 |
| Lumina | ρ | 0,94* | 1,00 | 0,93** |
|  | N | 11 | 12 | 12 |
| Microplate reader | ρ | 0,93* | 0,93** | 1.00 |
|  | N | 11 | 12 | 12 |

* Correlation is significant at the 0,05 level (2-tailed)

** Correlation is significant at the 0,01 level (2-tailed)
