# Supplementary material for: Visualisation and Quantitative Analysis of the Rodent Malaria Liver Stage by Real Time Imaging
Source: PLoS One. 2009 Nov 18;4(11):e7881. doi: 10.1371/journal.pone.0007881 (PMC2775639; doi:10.1371/journal.pone.0007881)
Supplement: Supplementary Table S2 — (0.06 MB DOC) [file pone.0007881.s002.doc]

**Supplementary Table S2**

Correlation coefficient data (ρ, two-tailed Spearman’s rho test) of the luminescence data (Lumina and Microplate reader) and the RT-qPCR data presented in Figure 2E.

|  |  | RTqPCR | Lumina | |
| --- | --- | --- | --- | --- |
|  |  |  | Whole body | Extracted liver |
| RTqPCR | ρ | 1,00 | 0,95** | 0,65* |
|  | N | 16 | 15 | 11 |
| Lumina  whole body | ρ | 0,95** | 1,00 | 0,80** |
|  | N | 15 | 15 | 11 |
| Lumina  extracted liver | ρ | 0,65* | 0,80** | 1,00 |
|  | N | 11 | 11 | 11 |

* Correlation is significant at the 0,05 level (2-tailed)

** Correlation is significant at the 0,01 level (2-tailed)
