# Supplementary figures and images for: Visualisation and Quantitative Analysis of the Rodent Malaria Liver Stage by Real Time Imaging
Source: PLoS One. 2009 Nov 18;4(11):e7881. doi: 10.1371/journal.pone.0007881 (PMC2775639; doi:10.1371/journal.pone.0007881)

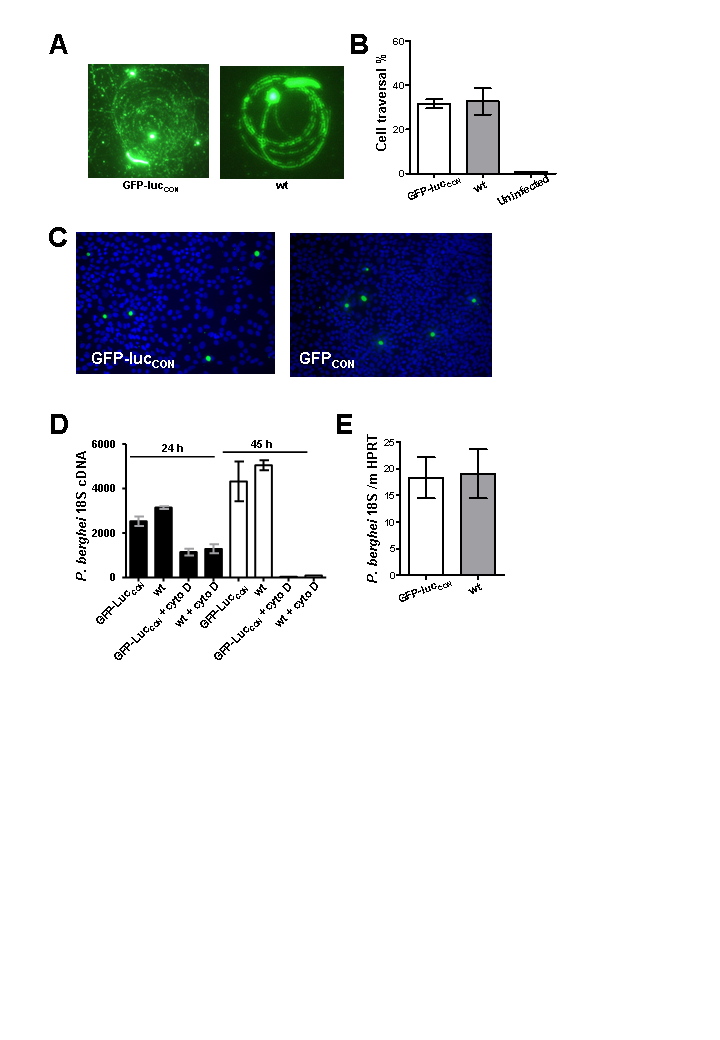

Supplement: Supplementary Figure S1 — Analyis of sporozoite motility, cell traversal and infectivity of PbGFP-Luccon A. Representative immunofluorescence staining with anti-PbCSP ([61]) of the trails produced by PbGFP-Luccon (left) and wild type sporozoites (right). Characteristic circles of gliding motility are observed in PbGFP-Luccon sporozoites. B. Cell traversal ability of wild type and PbGFP-Luccon sporozoites as determined by FACS counting of Dextran positive Huh7 cells. FACS counting was performed 3 h after infection of Huh7 cells with 6×10^4^ sporozoites. Uninfected: hepatocytes cultured in the presence of Dextran but without the addition of sporozoites. C. Infection of Huh7 cells on coverslips using 3×10^4^ PbGFP-Luccon (left) and PbGFPcon [39] (right) sporozoites. After fixing and staining, similar numbers of exoerythrocytic forms are observed at 48 h post infection for both parasites. D. qRT-PCR quantification of in vitro invasion of HepG2 cells by wild type and PbGFP-Luccon at 24 h (black bars) and at 45 h post invasion (white bars). Cyto D: cultures with cytochalasin-D. E. qRT-PCR quantification of liver invasion in mice of wild type and PbGFP-Luccon sporozoites. qRT-PCR was performed on material from livers collected at 43 h after infection of the mice with 3×10^4^ sporozoites. The pre-patent period, defined as the days between injection of sporozoites and a blood infection with a parasitemia of 0.5–2%, was 4.2 days (range 4–5 days) for PbGFP-Luccon compared to 4.4 days (range 4–5) for wild type parasites after injection of 1×10^4^ sporozoites. After injection of 1×10^4^ sporozoites the pre-patent periods were 5.3 days (range 5–6) for PbGFP-Luccon and 5.5 days (range 5–6) for wild type parasites. (0.28 MB TIF) [file pone.0007881.s003.tif]

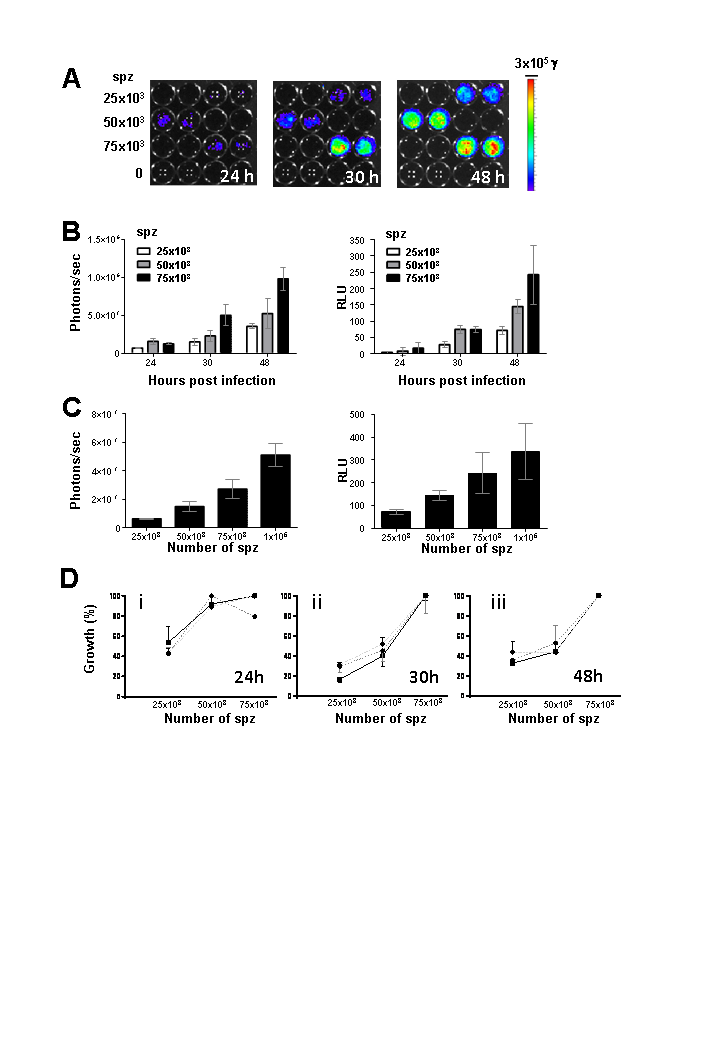

Supplement: Supplementary Figure S2 — Analysis of in vitro liver stage development in HepG2 cells by determination of luciferase expression (luminescence). A. Relationship between the numbers of sporozoites used to infect hepatocyte cultures and the luminescence produced by the liver stages at 24, 30 and 48 h after infection. Luminescence levels were measured using the Lumina system (Photons/sec). B. Relationship between the numbers of sporozoites used to infect hepatocyte cultures and the luminescence produced by the liver stages at 24, 30 h, 48 h after infection. Luminescence levels were measured using the Lumina system (Photons/sec) and a Wallac microplate reader (Relative light units, RLU), respectively. C. Relationship between the numbers of sporozoites used to infect hepatocyte cultures and the luminescence produced by the liver stages at 30 after infection. Luminescence levels were measured using the Lumina system (Photons/sec) and a Wallac microplate reader (Relative light unit, RLU), respectively. D. Correlation between luminescence values and 18S rRNA levels. Luminescence values were determined using the Lumina system and the Wallac microplate reader (see C). P. berghei 18S rRNA levels were determined by qRT-PCR of hepatocyte cultures infected with different numbers of sporozoites. The percentage of growth is normalized to the highest reading within each experiment. See Table S1 for the correlation coefficient data of the two-tailed Spearman's rho test. (0.15 MB TIF) [file pone.0007881.s004.tif]

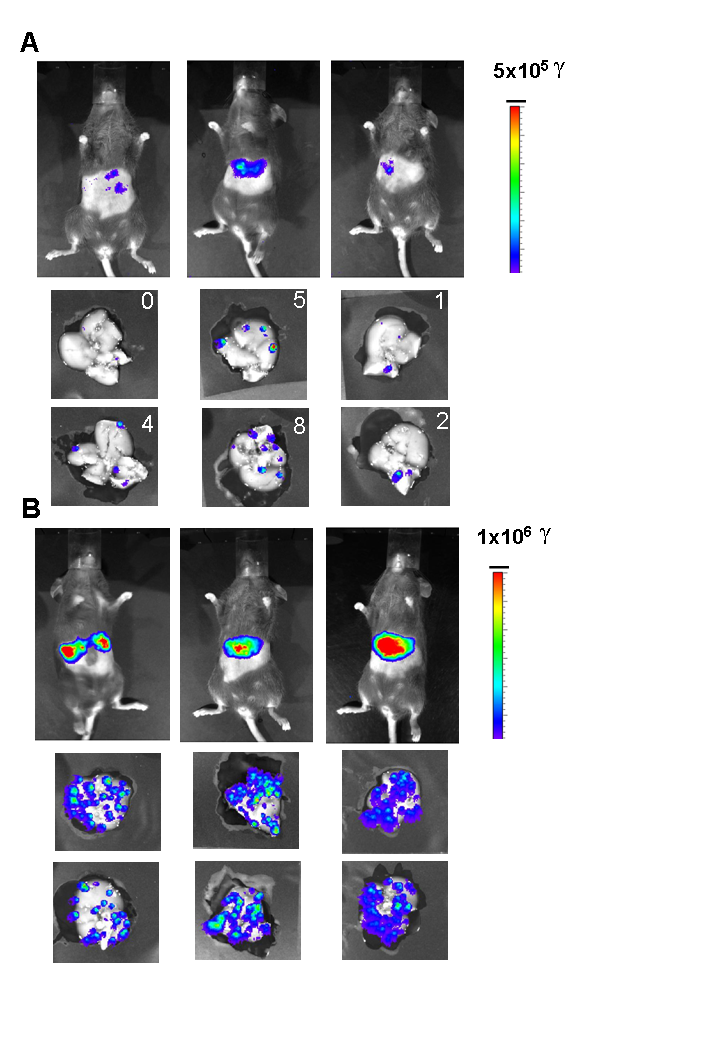

Supplement: Supplementary Figure S3 — Imaging of whole bodies and dissected livers (IVIS100) of mice at 44 h after infection by 1×10^3^ (A) or 1×10^4^ sporozoites (B). Dissected livers were imaged at both sides. Numbers in the pictures of Panel A show the number of luminescent spots identified. (0.68 MB TIF) [file pone.0007881.s005.tif]

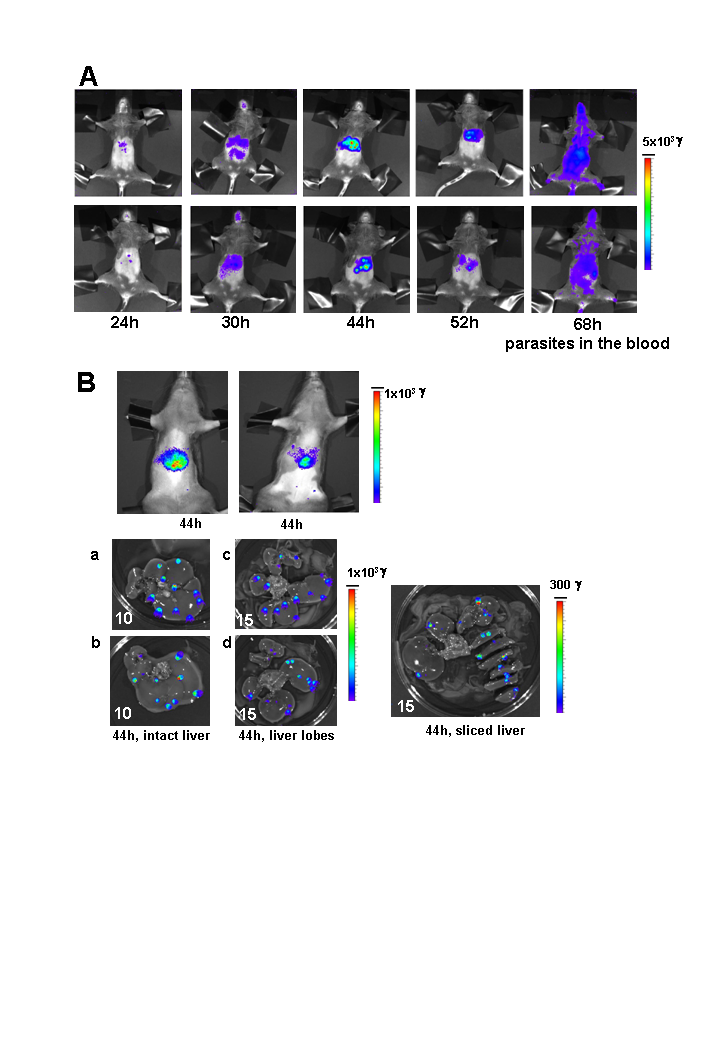

Supplement: Supplementary Figure S4 — A. Whole body imaging (IVIS100) of two representative mice during the period of 24–68 h after infection by bites of 20 infected mosquitoes, showing a strong increase of luminescence intensity of the liver during the period of 30–44 h after infection and a subsequent decrease after 52 h in the liver. The strong increase in luminescence of the whole body at 68 h is the result of the dissemination of the liver merozoites released into the bloodstream and subsequent invasion of erythrocytes. Rainbow images show the relative level of luminescence ranging from low (blue), to medium (green), to high (yellow/red). B. Imaging of whole bodies and extracted livers (IVIS100) of Wistar rats at 44 h after infection by bites of 1 or 5 infected mosquitoes. Extracted livers were measured at both sides (a, b) and lobes (c) and small sliced liver pieces (d) were analysed for additional luminescence spots. Numbers in the images represent the number of luminescent spots identified. (0.46 MB TIF) [file pone.0007881.s006.tif]

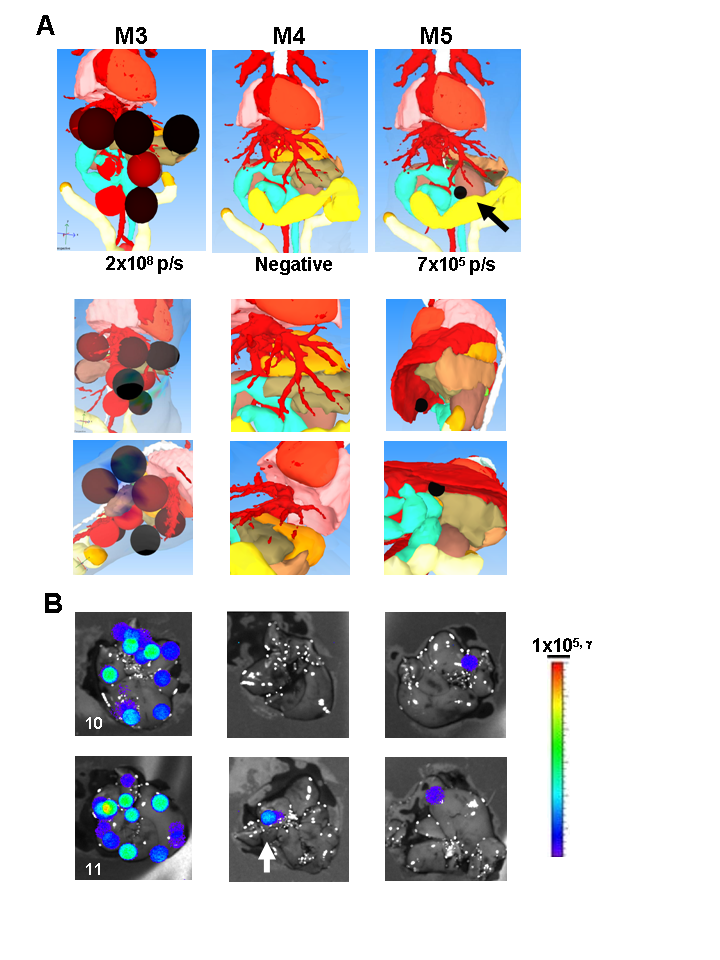

Supplement: Supplementary Figure S5 — A. Source reconstruction of 3D whole body imaging of three mice at 44 h after infection by bites of 5–10 infected mosquitoes. Eleven luminescent sources are detected in mouse 3 (M3), one in mouse 5 (M5) and none in mouse 4 (M4). See also Supplementary Movies 2 and 3 corresponding to mouse 3 and 5 respectively. B. 2D-imaging of the extracted livers of the mice shown in panel A. Livers were imaged at both sides using the IVIS Spectrum system. Numbers in the images represent the number of luminescent spots identified. Rainbow images show the relative level of luminescence ranging from low (blue), to medium (green), to high (yellow/red). (0.74 MB TIF) [file pone.0007881.s007.tif]
